# Unsupervised Machine Learning in Identification of Septic Shock Phenotypes and Their In-Hospital Outcomes: A Multicenter Cohort Study

**DOI:** 10.3390/jcm14134450

**Published:** 2025-06-23

**Authors:** Song Peng Ang, Jia Ee Chia, Eunseuk Lee, Maria Jose Lorenzo-Capps, Madison Laezzo, Jose Iglesias

**Affiliations:** 1 Department of Medicine, Division of Cardiology, Sarver Heart Center, University of Arizona College of Medicine, Tucson, AZ 85724, USA; 2 Department of Medicine, Rutgers Health/Community Medical Center, Toms River, NJ 08755, USAmaria.lorenzocapps@rwjbh.org (M.J.L.-C.); 3 Department of Medicine, Texas Tech University Health Science Center, El Paso, TX 79905, USA; 4 Department of Medicine, Hackensack Meridian School of Medicine, Nutley, NJ 07110, USA

**Keywords:** septic shock, phenotypes, biomarkers, cluster analysis, machine learning, outcomes, mortality, multicenter

## Abstract

**Background:** Septic shock is a heterogeneous syndrome with diverse clinical presentations and pathophysiology, yet current management guidelines largely treat it as a homogenous entity. Early risk stratification relies on lactate and different predictive scoring systems, which may not capture the underlying heterogeneity in host responses. **Aim:** To identify discrete subphenotypes of septic shock using admission demographics and laboratory parameters, and to evaluate their relationship with in-hospital outcomes. **Methods:** We conducted a retrospective multicenter cohort study of 10,462 adult patients with ICD-10-defined septic shock admitted to intensive care units between 2014 and 2015. We used Two-Step Cluster Analysis using log-likelihood distance and the Bayesian Information Criterion to identify two distinct phenotypes. We compared clusters on baseline characteristics, in-hospital outcomes including mortality, days on mechanical ventilation, vasopressor use, acute kidney injury (AKI), AKI requiring renal replacement therapy (RRT), and ICU and hospital lengths of stay. **Results:** We identified two clusters (Cluster 1, n = 5355 and Cluster 2, n = 5107) in our study. Cluster 1 showed greater biochemical severity at presentation, including higher median lactate (2.40 vs. 2.20 mmol L^−1^; *p* < 0.001), serum creatinine (1.39 vs. 1.20 mg dL^−1^; *p* < 0.001), blood urea nitrogen (28 vs. 25 mg dL^−1^; *p* < 0.001), and neutrophil-to-lymphocyte ratio (11.12 vs. 10.38; *p* < 0.001), and a higher mean SOFA score (7.05 ± 3.85 vs. 6.76 ± 3.87; *p* < 0.001). Despite this, Cluster 1 required mechanical ventilation more frequently (46.1% vs. 42.2%; *p* < 0.001) and had a higher incidence of AKI (58.1% vs. 55.6%; *p* = 0.009), including more stage 3 AKI (17.2% vs. 15.2%; *p* < 0.001) and dialysis (6.6% vs. 5.2%; *p* = 0.005), yet experienced similar in-hospital mortality (15.4% vs. 15.8%; *p* = 0.615) and comparable ICU (2.18 vs. 2.26 days; *p* = 0.254) and hospital lengths of stay (6.63 vs. 6.80 days; *p* = 0.251). **Conclusions:** Two septic shock phenotypes were identified, one with marked early organ dysfunction (Cluster 1) and another with milder initial derangements (Cluster 2), yet both showed convergent short-term mortality and lengths of stay despite divergent support needs. These results challenge reliance on single-parameter severity markers and underscore the need for phenotype-guided risk stratification and personalized management strategies in septic shock.

## 1. Introduction

Sepsis is not a monolithic disease but rather a heterogeneous syndrome with diverse clinical presentations, underlying pathophysiological mechanisms, and outcomes [1,2]. This heterogeneity presents significant challenges for clinicians in terms of patient management and for researchers attempting to develop effective therapeutic interventions. The current clinical definitions and treatment guidelines for septic shock, while valuable, often treat patients as a homogeneous group, potentially masking important biological and clinical differences that could impact treatment responses and outcomes [3].

The selection of adequate indicators for tissue hypoxia and appropriate resuscitation strategies remains a highly relevant issue in shock management [4]. Current guidelines advocate for the use of lactate as the primary metabolic marker, but this approach has recognized limitations [5]. Septic shock’s complex pathophysiology involves distributive shock mechanisms where cardiac output is shunted to peripheral tissues, potentially reducing oxygen delivery to vital organs even when peripheral oxygen saturation appears adequate. Of particular interest are ratios of leukocytes and platelets that may serve as biomarkers of the level of the inflammatory state [6,7,8,9]. Additionally, the lactate/albumin ratio, a biomarker of tissue metabolic dysregulation and acute inflammation, has shown promise as a predictor of mortality in various conditions.

The identification of phenotypes in critical illness has increasingly demonstrated tangible clinical value by enabling precision medicine approaches that extend beyond the uniform treatment protocols toward targeted, phenotype-guided therapy [10,11]. In sepsis, reproducible hyperinflammatory and hypoinflammatory subphenotypes have shown differential treatment responses. For instance, subanalysis of the PROWESS SHOCK trial (ClinicalTrials.gov number, NCT00604214) showed that hyperinflammatory patients derived survival benefit from activated protein C, while the counterpart hypoinflammatory phenotype patients did not [10,12]. Similarly, pediatric sepsis endotype classification may guide corticosteroid use in this population, based on combined blood protein signatures for assessment of mortality risk and whole blood gene expression profiling [13,14]. These examples illustrate the potential for subphenotype recognition to inform clinical decision-making, encourage individualized therapy selection, and ultimately enable precision approaches that target specific biologic mechanisms driving each patient’s unique disease trajectory rather than applying a one-size-fits-all management approach.

In recent years, machine learning techniques have emerged as powerful tools for identifying distinct subphenotypes within heterogeneous syndromes like sepsis [1,15]. Unsupervised clustering methods have been successfully employed to identify clinically meaningful patient subgroups based on clinical and biological parameters [16]. These approaches potentially offer distinct yet novel insights into disease mechanisms, therapeutic targets, and biomarkers essential for developing phenotype-driven therapies.

Despite these advances, important gaps remain in our understanding of septic shock subphenotypes. Many previous studies have focused on specific organ dysfunctions or risk trajectories, while fewer have examined the relationship between demographics, comorbidities, and laboratory parameters in defining septic shock clusters. Therefore, this study aims to identify distinct subphenotypes of septic shock patients based on demographic characteristics, comorbidities, and laboratory parameters, with a particular focus on leukocyte platelet ratios and lactate-to-albumin ratios and the association of these phenotypes and in-hospital outcomes.

## 2. Methods

**Figure 1:**
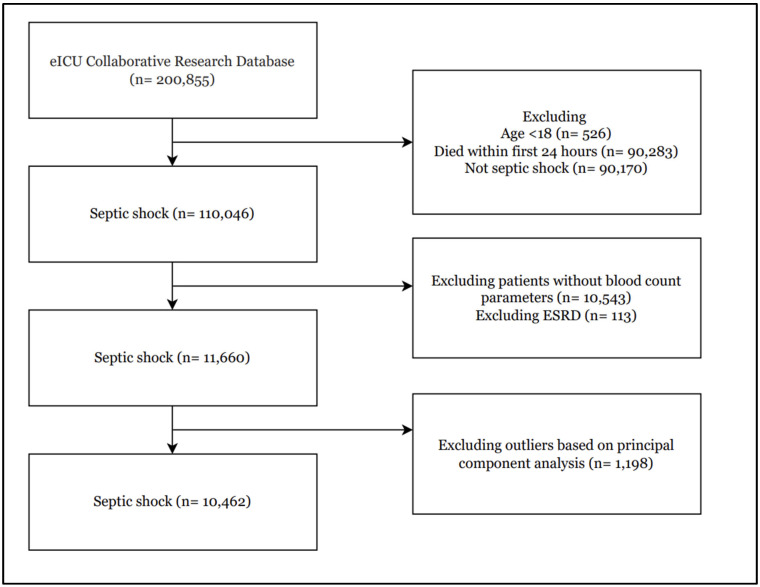
Flow diagram of patient selection.

We conducted a retrospective, multicenter cohort study using the eICU Collaborative Research Database, which collects de-identified patient data from over 200 intensive care units across the United States, on data collected between 2014 and 2015 [17]. Septic shock was identified using the International Classification of Diseases, 10th Revision, Clinical Modification (ICD-10-CM) codes. We first excluded patients under 18 years of age and those receiving chronic dialysis or with end-stage renal disease to focus on de novo septic shock physiology. Next, we removed records lacking baseline hematologic parameters required for clustering, as well as statistical outliers identified during principal component analysis to ensure the robustness of our phenotype derivation. Details of patient selection are available in Figure 1. The study is exempt from institutional review board approval due to its retrospective design, lack of direct patient intervention, and the security schema, for which the re-identification risk was certified as meeting safe harbor standards by an independent privacy expert (Privacert, Cambridge, MA, USA) (Health Insurance Portability and Accountability Act Certification no. 1031219-2) [17].

### 2.1. Study Variables and Outcomes

Demographic variables (age, sex, race), comorbidities, and initial laboratory parameters were extracted from electronic health records during the ICU hospitalization. Outcomes include overall mortality, ICU and hospital length of stay, days on mechanical ventilation, acute kidney injury (AKI), and AKI requiring renal replacement therapy (RRT). AKI is defined based on the 2012 Kidney Disease: Improving Global Outcomes (KDIGO) criteria [18].

### 2.2. Statistical Analysis

We implemented an unsupervised machine learning approach specifically designed to identify latent septic shock phenotypes without prior outcome knowledge. The methodology consisted of two sequential phases: dimensionality reduction through principal component analysis (PCA) followed by unsupervised clustering. The PCA was performed using the following standardized variables obtained on admission: neutrophil/lymphocyte ratio, monocyte/lymphocyte ratio, platelet/lymphocyte ratio, alanine aminotransferase (ALT), aspartate aminotransferase (AST), total bilirubin, serum sodium, serum potassium, serum magnesium, serum chloride, hemoglobin, age, and Sequential Organ Failure Assessment (SOFA) score. We evaluated the scree plot break point (elbow) to select the number of important principal components. The correspondence of data was calculated using the principal factors that were identified by PCA-transformed data. The Kaiser–Meyer–Olkin (KMO) test and Bartlett’s test of Sphericity were used to assess the adaptive validity of the PCA. The representative variables of the principal components were chosen based on their factor loading.

**Figure 2:**
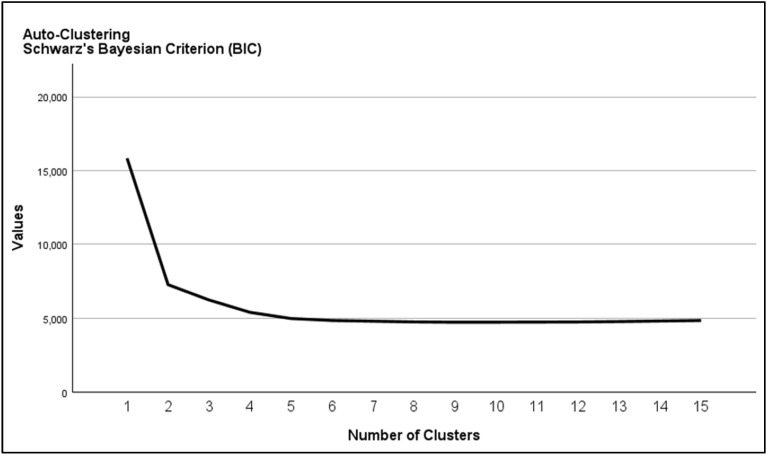
Bayesian Information Criterion (BIC) plot.

Following that, we implemented a Two-Step Cluster Analysis to identify natural groupings within the dataset of septic shock patients. This scalable clustering algorithm was selected for its ability to handle categorical variables and automatically determine the optimal number of clusters [19]. The log-likelihood distance measure was applied, assuming variable independence and multinomial distributions. Automatic cluster determination used the Bayesian Information Criterion (BIC) to identify optimal solutions (Figure 2). The number of clusters was determined by analyzing BIC change ratios at each successive merger relative to the first. Categorical variables were summarized as frequencies and percentages and compared between clusters using the χ^2^ test or Fisher’s exact test when any expected cell count was <5. Continuous variables were assessed for normality by the Shapiro–Wilk test; normally distributed variables are reported as mean ± standard deviation and compared with the Student’s *t*-test, whereas non-normally distributed variables are reported as median (interquartile range) and compared using the Mann–Whitney U (Wilcoxon rank-sum) test. All statistical tests were two-sided, and a *p*-value < 0.05 was considered significant. All analyses were performed using IBM SPSS Statistics (Version 27).

## 3. Results

### 3.1. Principal Component Analysis

**Figure 3:**
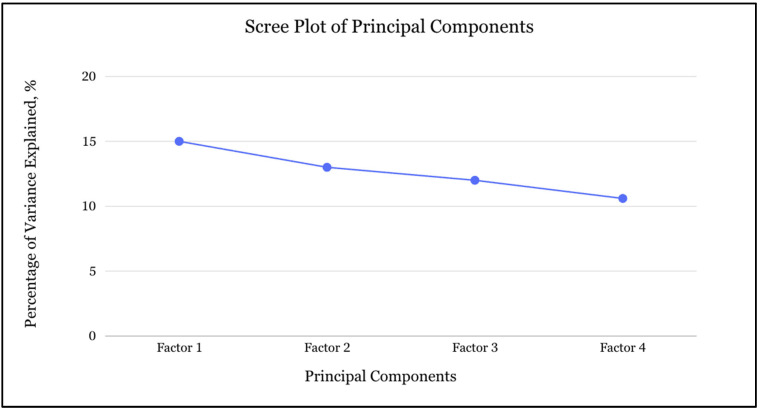
Scree plot of principal components.

The principal component analysis yielded four orthogonal dimensions with a KMO score of 0.55 and the Bartlett’s test of Sphericity result with a *p*-value of 0.0001 that together explain approximately 50.6% of the total variance in the current septic shock cohort (Figure 3, Table 1). Factor 1 (15% variance) is defined by strong positive loadings on lactic acid/albumin ratio (0.707), platelet-to-lymphocyte ratio (0.548), neutrophil-to-lymphocyte ratio (0.514), monocyte-to-lymphocyte ratio (0.509), and serum sodium (0.488), suggesting an inflammatory axis. Factor 2 (13% variance) is dominated by negative loadings on serum chloride (−0.865), ALT (−0.797), and total bilirubin (−0.693), indicating a hepatic dysfunction dimension, with patients scoring highly here tending to have deranged liver enzymes and bilirubin, along with chloride disturbances. Additionally, factor 3 (12% variance) loads almost exclusively on INR (0.918) and AST (0.911), pointing to a combination of coagulopathy and a hepatocellular injury component, in which clotting abnormalities and transaminase elevations jointly characterize a subgroup. Lastly, factor 4 (10.6% variance) shows very strong negative loadings on serum creatinine (−0.966) and age (−0.956), representing an age–renal factor: older patients with worse renal function cluster together along this axis.

### 3.2. Demographics and Clinical Comorbidities, by Clusters

In this retrospective cohort of 10,462 patients with septic shock, 5355 (51.2%) were assigned to Cluster 1 and 5107 (48.8%) to Cluster 2 (Figure 1, Table 2). Although the median age did not differ, Cluster 1 had predominantly male patients while Cluster 2 had predominantly female patients (*p* < 0.001). Ethnic composition also diverged (*p* = 0.001): Cluster 1 included higher proportion of African American (10.5% vs. 9.9%) and Other/Unknown (6.5% vs. 5.0%) patients, whereas Cluster 2 included higher proportions of Asian (2.3 % vs. 1.5%), Caucasian (76.4% vs. 75.4%), and Hispanic (5.8% vs. 5.3%) individuals.

Comorbidity burdens were largely similar, but hyperlipidemia (2.6% vs. 1.9%; *p* = 0.017) and chronic kidney disease (12.5% vs. 11.3%; *p* = 0.045) were more common in Cluster 2. No significant between-cluster differences were observed in cirrhosis, hypertension, diabetes, chronic obstructive pulmonary disease, cerebrovascular disease, malignancy, or heart failure (all *p* > 0.05). In keeping with these comorbidity patterns, prior medication use also differed: Cluster 2 patients were more often prescribed diuretics (17.6% vs. 15.4%; *p* = 0.002) and angiotensin receptor blockers (3.4% vs. 2.1%; *p* < 0.001), while angiotensin-converting enzyme inhibitor use was comparable (8.6% vs. 7.8%; *p* = 0.121).

### 3.3. Admission Characteristics, by Clusters

At baseline, the two data-driven phenotypes were demographically similar, with a median age of 68 years in both cohorts, yet their biochemical profiles differed in ways that portend differential physiological stress (Table 3). Cluster 2 presented with a marginally faster peak heart rate than Cluster 1 (median 112 bpm vs. 110 bpm; *p* = 0.003) and had larger circulating platelet and total leucocyte burdens, the latter driven chiefly by neutrophilia. By contrast, patients in Cluster 1 had relative thrombocytopenia and lymphopenia, resulting in a higher neutrophil-to-lymphocyte ratio (median 11.12 vs. 10.38; *p* < 0.001) and greater monocyte-to-lymphocyte imbalance. Electrolyte analyses revealed subtly higher potassium and magnesium concentrations in Cluster 1, while sodium was lower in Cluster 2 (all *p* < 0.05). Markers of metabolic stress and organ dysfunction, however, were conspicuously worse in Cluster 1: lactate was higher (median 2.40 mmol L^−1^ vs. 2.20 mmol L^−1^; *p* < 0.001), as was the lactate-to-albumin ratio, and indices of renal impairment—including blood urea nitrogen (28 mg dL^−1^ vs. 25 mg dL^−1^; *p* < 0.001) and creatinine (median 1.39 mg dL^−1^ vs. 1.20 mg dL^−1^; *p* < 0.001)—were consistently greater. These biochemical derangements translated into a higher SOFA score in Cluster 1 (mean 7.05 ± 3.85 vs. 6.76 ± 3.87; *p* < 0.001) compared to Cluster 2.

### 3.4. In-Hospital Events and Outcomes

In-hospital mortality was comparable between the two phenotypes, affecting 15.4% of Cluster 1 and 15.8% of Cluster 2 patients (*p* = 0.615) (Table 4). Acute kidney injury occurred more frequently in Cluster 1 (58.1% vs. 55.6%; *p* = 0.009), and the distribution of AKI stages differed significantly (*p* < 0.001): Cluster 1 had fewer AKI-free patients (41.9% vs. 44.4%) and a greater proportion with severe injury including stage 3 AKI (17.2% vs. 15.2%) and a higher requirement for RRT (6.6% vs. 5.2%; *p* = 0.005). Despite these differences in renal support requirement, the median duration of mechanical ventilation was comparable in both groups (3 [2–6] days; *p* = 0.221), as were intensive care unit length of stay (2.18 vs. 2.26 days; *p* = 0.254) and overall hospital length of stay (6.63 vs. 6.80 days; *p* = 0.251).

## 4. Discussion

In our cohort of 10,462 septic shock patients, Cluster 1 presented with markedly higher admission severity, including elevated lactate, creatinine, neutrophil-to-lymphocyte ratio, and SOFA scores, whereas Cluster 2 exhibited comparatively milder initial laboratory derangements. Despite this, the rate of in-hospital mortality, length of hospital stay, and length of ICU stay were similar between both clusters of patients. Cluster 1 had a higher incidence of AKI and AKI requiring RRT compared to Cluster 2.

### 4.1. Comparison with Prior Sepsis Phenotypes

Our two-cluster model reveals a dichotomy reminiscent of previously described sepsis phenotypes [20]. Cluster 1 patients presented with more overt shock and organ dysfunction, as evidenced by high lactate, elevated creatinine, a high neutrophil–lymphocyte ratio (NLR), and greater SOFA scores. This profile aligns with the “shock with elevated creatinine” subtype identified by Knox et al. [21]. In a study by Knox et al., their Cluster 1 similarly consisted of patients with acute kidney dysfunction, yet the in-hospital mortality was strikingly low at approximately 11%. Our findings reflected this pattern, whereby despite greater initial severity, Cluster 1 did not experience worse outcomes than Cluster 2. Nevertheless, these findings were different from those of the study by Seymour et al. [22]. Seymour and colleagues investigated a total of 20,189 patients from three observational cohort studies and three randomized trials. They found that the phenotype characterized by high lactate, hepatobiliary injury, and shock (“δ” phenotype) had the highest mortality. The discrepancy may stem from differences in cohort case-mix and phenotype definitions. Notably, our Cluster 1’s combination of lactate-driven shock and acute kidney injury resembles features of Seymour’s “δ” (shock/liver) and “β” (renal dysfunction) phenotypes [22]. However, the outcomes of our clusters seem more in line with Knox et al.’s observations, where an observed severe shock phenotype did not translate into excess mortality [21].

Cluster 2 patients, in contrast, had lower initial lactate and milder initial lab abnormalities, akin to Seymour’s “β phenotype” (older patients with fewer extreme lab abnormalities, often with chronic comorbidities) or Knox’s “minimal MODS” group. Such patients may appear less critically ill on presentation. However, importantly, these "milder" subsets of patients did not always translate into better outcomes. For instance, in Seymour’s study, the β phenotype had a comparable mortality of 13%, much higher than the most benign α phenotype within the study (5% mortality). Our Cluster 2 mirrors the findings of these prior studies. Despite initial lower severity scores, its rates of mechanical ventilation, acute kidney injury, and need for renal replacement therapy were not lower than those of Cluster 1. This suggests that Cluster 2 represents the “clinically quiescent at onset yet intrinsically high-risk” phenotype. In summary, Cluster 1 and Cluster 2 appear to recapitulate known sepsis subgroups: one akin to a hyper-acute shock phenotype (high lactic acidosis, hyperinflammatory) and the other a less fulminant, perhaps more chronic or insidious phenotype.

The current study demonstrates that divergent initial phenotypes converge in mortality and resource utilization, which has implications for the timing of interventions and resource utilization. In contrast to the work of Seymour et al. and Knox et al., whose studies focused on heterogeneous sepsis populations, potentially underrepresenting patients with septic shock, the current work specifically focuses on patients with septic shock. These critically ill patients have higher resource utilization and mortality. Additionally, septic shock accounts for a high percentage of sepsis-related mortality, whose cause remains poorly understood and characterized in terms of early phenotypes. The current study demonstrates that even in this high-risk group, significant heterogeneity exists. Unlike Seymour et al., who used dynamic variables obtained after 24 hrs, we derived the current clusters from laboratory data obtained at admission, which provides a real-time phenotype-driven risk assessment during the hour of resuscitation [22]. Interestingly, our data demonstrate very few differences in mortality, which is a potential signal identifying a silent risk phenotype.

It would also be instructive to consider molecular endotypes in the context of our clusters [23,24,25]. Gene expression studies have repeatedly identified several major sepsis endotypes, including one that is immunologically dysregulated and high-risk, and the other that is more immunocompetent, with better outcomes [26]. Wong et al. described pediatric septic shock endotype A, which is associated with adaptive immune suppression and corticosteroid unresponsiveness, while endotype B is associated with more intact immunity and lower mortality [23]. In our study, Cluster 2 may represent an immunosuppressed endotype analogous to endotype A. Conversely, Cluster 1’s robust inflammatory presentation might reflect an immune-responsive endotype akin to endotype B, capable of mounting a strong defense and recovering. This interpretation fits the paradox observed and shows that immunophenotype-driven risk may be a supplementary index to the bedside severity metrics. Indeed, prior work showed that assignment to endotype A carried a higher mortality and organ failure burden even after controlling for illness severity.

### 4.2. Implications of Future Risk Stratifications and Treatment Strategies

Our findings highlight the potential of phenotype-based risk stratification in septic shock. Traditional markers, including lactate and SOFA, captured acute severity but did not fully predict outcomes in this heterogeneous cohort. Recognizing a patient’s sepsis phenotype or endotype could augment prognostic accuracy and guide individualized treatment strategies. Prior studies have shown that phenotype-based approaches in sepsis can identify subgroups with differing outcomes and treatment responses. For example, Seymour et al. demonstrated that early-recognized clinical phenotypes were associated with reproducible differences in mortality, organ dysfunction, and biomarker profiles, and influenced the interpretation of trial results [22]. Others have shown that these phenotypes can guide resuscitation strategies, immunomodulatory therapies, and triage decisions [10,27,28].

If Cluster 1 represents a hyperinflammatory shock subtype, these patients might benefit most from rapid hemodynamic support, source control, and perhaps therapies targeting the fulminant inflammatory response, such as early use of vasoactive drugs. Their relatively favorable outcomes suggest that current standard care is effective, but phenotype-specific interventions, such as tailored fluid dosing or vasopressor selection, could further optimize outcomes.

Likewise, a patient in Cluster 2, with initially lower lactate and SOFA scores, should not be under-triaged. In fact, they may still actually carry a high risk for deterioration, as evidenced by their comparable rates of mechanical ventilation, acute kidney injury, and need for renal replacement therapy despite appearing less severely ill at presentation. Integrating such phenotype recognition into early warning scores or ICU triage protocols could help allocate resources more wisely, ensuring that “low- or silent-risk” patients (Cluster 2) receive similar intensified monitoring, while truly low-risk patients avoid unnecessary interventions. Recent reviews have also emphasized that such phenotype-based approaches are increasingly feasible and may enhance precision in both clinical care and future trial design [29,30].

### 4.3. Strength and Limitations

The strength of our findings lies in the study’s use of an extensive database from a multicenter critical care database, making it well-suited for the application of unsupervised learning techniques. Machine learning facilitates the detection of subtle regularities and relationships between subgroups in large databases with heterogeneous clinical data, which may not be detected with traditional supervised machine learning or other analytical methods. A major setback encountered with these tools is the lack of universally accepted methods for validating the findings. Thus, in contrast to supervised learning, they pose no predefined clinical outcome or methods of validation and are prone to introducing bias. Therefore, our findings should be considered theoretical and hypothesis-generating [31]. In terms of other limitations, its retrospective design and reliance on routinely collected electronic health-record data introduce the potential for misclassification and unmeasured confounding. Clustering was based solely on admission laboratory and demographic variables. Data on microbiology, source control timing, or detailed changes in laboratory parameters were not included in the clustering model. Second, we applied unsupervised clustering within this multicenter ICU cohort, and external validation in other populations, particularly in non-United States or non-academic settings, is needed to assess the generalizability of our phenotypes. Finally, as with all clustering approaches, the choice of variables, distance metrics, and number of clusters can affect the resulting phenotype [32]. Therefore, our two-cluster solution should be viewed as one among several plausible, hypothesis-generating representations of septic shock heterogeneity, rather than a definitive classification. While supervised validation using our clustering variables would create methodological circularity, future research should investigate the prognostic utility of these phenotypes through independent validation cohorts and novel biomarkers not used in cluster derivation. Additionally, these identified phenotypes could serve as predictive features in supervised machine learning models designed to forecast clinical outcomes, representing a complementary approach to our exploratory unsupervised methodology.

## 5. Conclusions

In this multicenter cohort of 10,462 patients with septic shock, unsupervised clustering of admission demographics and laboratory parameters delineated two robust phenotypes with distinct biochemical and clinical profiles but convergent short-term outcomes. Cluster 1 comprised patients with marked metabolic derangement, yet despite this apparent severity, their in-hospital mortality, duration of mechanical ventilation, and lengths of stay were similar to those of Cluster 2. By contrast, Cluster 2 exhibited milder initial laboratory abnormalities but demonstrated equal or higher rates of organ support, suggesting a phenotype of “silent risk” characterized by relative immune suppression or a limited physiological reserve. These findings challenge the assumption that early biochemical severity uniformly portends a poor prognosis, emphasizing the need to explore the potential of incorporating phenotype recognition into risk stratification and management algorithms. Personalized interventions ranging from aggressive hemodynamic optimization in hyperinflammatory shock to immunomodulatory or intensified monitoring strategies in the insidious phenotype may improve outcomes by targeting the underlying pathophysiology rather than relying solely on global severity scores. Prospective validation of these clusters and incorporation of molecular endotyping will be essential to translate phenotype-driven approaches into clinical practice and to refine our understanding of septic shock heterogeneity.

## Figures and Tables

**Table 1:** Principal component analysis.

Factors	1	2	3	4
Eigen value	2.17	2	2	1.6
Percent variance	15	13	12	10.6
Lactic acid/albumin	0.707	0.073	0.1	−0.017
Platelet/lymphocyte ratio	0.548	0.04	0.039	−0.066
Neutrophil/lymphocyte ratio	0.514	−0.044	−0.043	−0.02
Monocyte/lymphocyte ratio	0.509	0.06	−0.135	−0.035
Serum sodium	0.488	−0.034	0.072	0.039
Serum potassium	−0.362	0.027	0.05	−0.143
Hemoglobin	0.358	−0.007	0.023	−0.109
Serum chloride	0.01	−0.865	0.005	−0.037
Alanine aminotransferase	−0.074	−0.797	−0.022	−0.01
Total bilirubin	0.015	−0.693	−0.023	−0.015
INR	−0.073	0.123	0.918	−0.05
Aspartate aminotransferase	−0.013	0.129	0.911	0.006
SOFA score	0.06	−0.18	0.377	0.047
Serum creatinine	0.006	−0.037	−0.016	−0.966
Age	0.064	−0.033	−0.015	−0.956

SOFA = Sequential Organ Failure Assessment; INR = international normalized ratio.

**Table 2:** Demographics and clinical comorbidities of patients, by clusters.

Variables	Cluster 1 (N = 5355)	Cluster 2 (N = 5107)	*p*
Male gender	5355 (100)	-	<0.001
Ethnicity			0.001
African American	563 (10.50)	505 (9.90)	
Asian	83 (1.50)	118 (2.30)	
Caucasian	4040 (75.40)	3903 (76.40)	
Hispanic	286 (5.30)	295 (5.80)	
Native American	32 (0.60)	30 (0.60)	
Other/Unknown	350 (6.50)	256 (5.00)	
Comorbidities			
Cirrhosis	141 (2.70)	115 (2.30)	0.209
Hypertension	513 (9.60)	442 (8.70)	0.101
Diabetes	128 (2.40)	136 (2.70)	0.374
COPD	499 (9.30)	451 (8.80)	0.386
HLD	140 (2.60)	98 (1.90)	0.017
Cerebrovascular diseases	96 (1.80)	95 (1.90)	0.797
Malignancy	300 (5.60)	269 (5.30)	0.45
CKD	672 (12.50)	576 (11.30)	0.045
Heart failure	523 (9.80)	493 (9.70)	0.845
Prior medication use			
Diuretics	823 (15.40)	899 (17.60)	0.002
ACEI	463 (8.60)	399 (7.80)	0.121
ARB	115 (2.10)	172 (3.40)	<0.001

Variables expressed as frequency (%). ACEI: angiotensin-converting enzyme inhibitor; ARB: angiotensin receptor blocker; COPD: chronic obstructive pulmonary disease; CKD: chronic kidney disease; HLD: hyperlipidemia.

**Table 3:** Initial clinical and laboratory findings of patients, by clusters.

Variables	Overall N	Overall Mean ± SD	Overall Median (IQR)	Cluster 1 N	Cluster 1 Mean ± SD	Cluster 1 Median (IQR)	Cluster 2 N	Cluster 2 Mean ± SD	Cluster 2 Median (IQR)	*p*
Age, y	10,462	66.41 ± 16.26	68.00 (57.00–79.00)	5355	66.32 ± 15.85	68.00 (57.00–79.00)	5107	66.49 ± 16.68	68.00 (56.00–80.00)	0.205
Highest HR, bpm	9642	112.81 ± 27.82	111.00 (96.00–126.25)	4946	112.08 ± 28.00	110.00 (96.00–126.00)	4696	113.58 ± 27.62	112.00 (97.00–128.00)	0.003
Lowest MAP, mm Hg	1572	57.18 ± 15.01	58.00 (51.00–64.00)	816	56.97 ± 14.50	58.00 (50.67–64.33)	756	57.42 ± 15.56	57.67 (51.33–63.67)	0.807
Lowest temp, °F	10,019	97.22 ± 3.33	97.50 (96.80–98.10)	5132	97.21 ± 2.88	97.50 (96.80–98.10)	4887	97.22 ± 3.75	97.50 (96.80–98.10)	0.339
White blood cells, cells/µL	10,462	14,929.12 ± 10,370.37	13,500.00 (8975.00–19,000.00)	5355	14,683.10 ± 10,846.57	13,300.00 (8800.00–18,700.00)	5107	15,187.09 ± 9840.81	13,800.00 (9100.00–19,300.00)	<0.001
Platelets, cells/µL	10,462	234648.63 ± 125504.03	215,000.00 (152,000.00–296,000.00)	5355	224190.10 ± 123206.06	202,000.00 (143,000.00–281,000.00)	5107	245615.04 ± 126959.19	229,000.00 (161,000.00–309,000.00)	<0.001
Lymphocytes, cells/µL	10,462	1415.26 ± 4668.02	952.55 (555.00–1576.00)	5355	1447.62 ± 6253.00	900.90 (524.00–1497.60)	5107	1381.33 ± 1908.45	1008.00 (592.00–1677.00)	<0.001
Neutrophils, cells/µL	10,462	11,920.22 ± 7585.13	10,736.48 (6636.00–15,750.00)	5355	11,645.92 ± 7279.65	10,530.00 (6532.00–15,471.00)	5107	12,207.85 ± 7883.22	11,040.00 (6758.80–16,065.00)	0.001
Monocytes, cells/µL	10,462	895.92 ± 844.92	759.70 (420.00–1176.12)	5355	907.28 ± 869.34	768.00 (424.00–1195.00)	5107	884.01 ± 818.44	748.00 (420.00–1160.00)	0.172
Neutrophil–lymphocyte ratio	10,462	15.57 ± 15.73	10.73 (5.64–19.63)	5355	15.94 ± 15.78	11.12 (5.86–21.00)	5107	15.18 ± 15.67	10.38 (5.40–18.50)	<0.001
Platelet–lymphocyte ratio	10,462	309.76 ± 297.46	222.91 (130.38–380.33)	5355	312.49 ± 299.44	223.51 (130.64–386.29)	5107	306.89 ± 295.36	221.69 (130.14–374.05)	0.372
Monocyte–lymphocyte ratio	10,462	0.98 ± 0.90	0.75 (0.40–1.25)	5355	1.03 ± 0.91	0.80 (0.43–1.33)	5107	0.93 ± 0.89	0.67 (0.38–1.17)	<0.001
Sodium, mEq/L	10,462	136.16 ± 6.39	136.00 (133.00–139.80)	5355	136.31 ± 6.43	136.00 (133.00–140.00)	5107	135.99 ± 6.34	136.00 (133.00–139.00)	0.016
Potassium, mEq/L	10,436	4.25 ± 0.88	4.10 (3.70–4.70)	5342	4.31 ± 0.85	4.20 (3.80–4.70)	5094	4.20 ± 0.91	4.10 (3.60–4.60)	<0.001
Magnesium, mg/dL	8438	1.81 ± 0.48	1.80 (1.50–2.10)	4291	1.85 ± 0.45	1.80 (1.60–2.10)	4147	1.78 ± 0.52	1.70 (1.50–2.00)	<0.001
Albumin, g/dL	9342	3.07 ± 0.73	3.10 (2.60–3.60)	4790	3.09 ± 0.73	3.10 (2.60–3.60)	4552	3.06 ± 0.73	3.10 (2.60–3.60)	0.047
Lactate, mmol/L	8990	3.01 ± 2.49	2.30 (1.40–3.70)	4611	3.10 ± 2.54	2.40 (1.50–3.80)	4379	2.91 ± 2.43	2.20 (1.40–3.60)	<0.001
INR (ratio)	8019	1.59 ± 1.16	1.20 (1.10–1.50)	4218	1.60 ± 1.16	1.22 (1.10–1.50)	3801	1.58 ± 1.17	1.20 (1.10–1.50)	<0.001
ALT, U/L	10,462	47.23 ± 89.64	25.00 (16.00–43.00)	5355	47.91 ± 88.70	26.00 (17.00–44.00)	5107	46.52 ± 90.61	24.00 (15.00–42.00)	<0.001
AST, U/L	10,462	64.49 ± 146.19	29.00 (19.00–53.00)	5355	64.01 ± 141.73	30.00 (20.00–54.00)	5107	65.00 ± 150.72	29.00 (19.00–52.00)	0.203
Bicarbonate, mEq/L	9689	23.85 ± 5.76	24.00 (21.00–27.00)	4940	23.99 ± 5.68	24.00 (21.00–27.00)	4749	23.71 ± 5.84	24.00 (20.00–27.00)	0.006
Chloride, mEq/L	10,462	100.11 ± 7.36	100.00 (96.00–104.00)	5355	100.24 ± 7.31	100.00 (96.00–104.00)	5107	99.98 ± 7.41	100.00 (96.00–104.00)	0.156
BUN, mg/dL	10,449	34.48 ± 26.10	27.00 (17.00–44.00)	5350	35.75 ± 26.45	28.00 (18.00–45.00)	5099	33.15 ± 25.66	25.00 (16.00–43.00)	<0.001
Lactate/albumin ratio	8033	1.08 ± 1.08	0.76 (0.47–1.28)	4116	1.10 ± 1.08	0.79 (0.49–1.30)	3917	1.06 ± 1.09	0.75 (0.45–1.25)	<0.001
SOFA score (points)	10,438	6.91 ± 3.86	7.00 (4.00–10.00)	5346	7.05 ± 3.85	7.00 (4.00–10.00)	5092	6.76 ± 3.87	7.00 (4.00–9.00)	<0.001
Serum creatinine, mg/dL	10,438	1.90 ± 1.82	1.30 (0.87–2.20)	5342	2.04 ± 1.95	1.39 (0.95–2.30)	5096	1.75 ± 1.66	1.20 (0.79–2.07)	<0.001

ALT = alanine aminotransferase; AST = aspartate aminotransferase; BUN = blood urea nitrogen; SOFA = Sequential Organ Failure Assessment.

**Table 4:** ICU management, in-hospital events and outcomes, by clusters.

Variables	Cluster 1 (N = 5355)	Cluster 2 (N = 5107)	*p*
Ventilation	2470 (46.10)	2154 (42.20)	<0.001
Vasopressor use	2103 (39.30)	2011 (39.40)	0.912
Mortality	825 (15.40)	805 (15.8)	0.615
AKI	3111 (58.10)	2837 (55.60)	0.009
Stages of AKI *			<0.001
0	2244 (41.90)	2270 (44.40)	
1	1748 (32.60)	1536 (30.10)	
2	441 (8.20)	526 (10.30)	
3	922 (17.20)	775 (15.20)	
AKI requiring RRT	351 (6.60)	268 (5.20)	0.005
Days on mechanical ventilation, days	3.00 (2.00–6.00)	3.00 (2.00–6.00)	0.221
Hospital LOS, days	6.63 (4.07–10.92)	6.80 (4.01–11.43)	0.251
ICU LOS, days	2.18 (1.21–4.05)	2.26 (1.21–4.21)	0.254

AKI: acute kidney injury; LOS: length of stay; RRT: renal replacement therapy. * based on Kidney Disease: Improving Global Outcomes (KDIGO) criteria.

## Data Availability

The data supporting this study were extracted from the eICU database. The data are publicly available at https://eicu-crd.mit.edu/, accessed on 10 March 2025.
